# *TP53* hotspot mutations are predictive of survival in primary central nervous system lymphoma patients treated with combination chemotherapy

**DOI:** 10.1186/s40478-016-0307-6

**Published:** 2016-04-22

**Authors:** Helga D. Munch-Petersen, Fazila Asmar, Konstantinos Dimopoulos, Aušrinė Areškevičiūtė, Peter Brown, Mia Seremet Girkov, Anja Pedersen, Lene D. Sjö, Steffen Heegaard, Helle Broholm, Lasse S. Kristensen, Elisabeth Ralfkiaer, Kirsten Grønbæk

**Affiliations:** Department of Hematology, Rigshospitalet, Copenhagen University Hospital, Department 3733, Copenhagen Biocenter, Building 2, 3rd floor, Ole Maaløes Vej 5, 2200 Copenhagen N, Denmark; Department of Pathology, Rigshospitalet, University Hospital of Copenhagen, Copenhagen, Denmark; Department of Ophtalmology, Rigshospitalet, University Hospital of Copenhagen, Copenhagen, Denmark

**Keywords:** PCNSL, *TP53*, *MIR34A*, Hotspot mutations, DNA methylation, Survival

## Abstract

Primary central nervous system lymphoma (PCNSL) is an aggressive variant of diffuse large B-cell lymphoma (DLBCL) confined to the CNS. *TP53* mutations (*MUT-TP53*) were investigated in the context of *MIR34A/B/C*- and *DAPK* promoter methylation status, and associated with clinical outcomes in PCNSL patients. In a total of 107 PCNSL patients clinical data were recorded, histopathology reassessed, and genetic and epigenetic aberrations of the p53-miR34-DAPK network studied. *TP53* mutational status (exon 5–8), with structural classification of single nucleotide variations according to the IARC-TP53-Database, methylation status of *MIR34A/B/C* and *DAPK,* and p53-protein expression were assessed. The 57/107 (53.2 %) patients that were treated with combination chemotherapy +/− rituximab (CCT-treated) had a significantly better median overall survival (OS) (31.3 months) than patients treated with other regimens (high-dose methotrexate/whole brain radiation therapy, 6.0 months, or no therapy, 0.83 months), *P* < 0.0001. *TP53* mutations were identified in 32/86 (37.2 %), among which 12 patients had hotspot/direct DNA contact mutations. CCT-treated patients with PCNSL harboring a hotspot/direct DNA contact *MUT-TP53* (*n* = 9) had a significantly worse OS and progression free survival (PFS) compared to patients with non-hotspot/non-direct DNA contact *MUT-TP53* or wild-type *TP53* (median PFS 4.6 versus 18.2 or 45.7 months), *P =* 0.041 and *P =* 0.00076, respectively. Multivariate Cox regression analysis confirmed that hotspot/direct DNA contact *MUT-TP53* was predictive of poor outcome in CCT-treated PCNSL patients, *P* = 0.012 and *P =* 0.008; HR: 1.86 and 1.95, for OS and PFS, respectively. *MIR34A, MIR34B/C,* and *DAPK* promoter methylation were detected in 53/93 (57.0 %), 80/84 (95.2 %), and 70/75 (93.3 %) of the PCNSL patients with no influence on survival. Combined *MUT-TP53* and *MIR34A* methylation was associated with poor PFS (median 6.4 versus 38.0 months), *P* = 0.0070. This study suggests that disruption of the p53-pathway by *MUT-TP53*in hotspot/direct DNA contact codons is predictive of outcome in CCT-treated PCNSL patients, and concomitant *MUT-TP53* and *MIR34A* methylation are associated with poor PFS.

**Electronic supplementary material:**

The online version of this article (doi:10.1186/s40478-016-0307-6) contains supplementary material, which is available to authorized users.

## Introduction

Primary central nervous system lymphoma (PCNSL) of the diffuse large B-cell lymphoma (DLBCL) subtype is an aggressive variant of DLBCL confined to the CNS [44]. Patients with extra CNS DLBCL display diverse clinical courses, which may be explained by considerable heterogeneity of the clinical, morphological and cytogenetic phenotypes [29, 37, 40, 52]. PCNSL or CNS DLBCL has specific features that further distinguish this subtype from DLBCL [11, 44]. Despite extensive research, the clinical outcome of patients diagnosed with PCNSL is still dismal, a fact that cannot only be explained by the specific anatomical site or the difficulty of drug-delivery across the blood-brain-barrier [14, 15, 21, 30, 32, 36, 39, 54]. The pathogenic mechanisms driving this disease are still not clear, and only few genetic or epigenetic aberrations have been suggested as predictive or prognostic markers in PCNSL [1, 29, 31].

The *TP53* tumor suppressor gene plays important roles in the regulation of cell proliferation, apoptosis and genomic integrity. Under stressful conditions, i.e. DNA damage, hypoxia, or oncogenic activation, the wild-type *TP53* (*WT-TP53*) gene product p53 mediates cell-cycle arrest and preserves genomic stability via transcription-dependent activity (TA) and transcription-independent activity (TIA) in the cell. Reversely, the mutant p53 protein may be non-functional or have gain-of-function properties both leading to genomic instability and uncontrolled proliferation of damaged cells [27, 33, 34, 43, 51].

In DLBCL outside the CNS, *TP53* mutations (*MUT-TP53*) have mostly been studied with emphasis on the disruption of the coding sequences of the DNA binding domain (DBD) (exons 5–8) with a reported mutation frequency of ~20–25 % [35, 52]. Structural classification and functional characteristics of *MUT-TP53* assessed by yeast-based functional assays, available in the International Agency for Research on Cancer (IARC)-TP53-Database (http://www.iarc.fr), have been reported in DLBCL, with worse prognosis associated with mutations in the DBD of *TP53* [35, 51, 52]. Indeed, the majority of cancer-related *TP53* mutations occur in the region of the DBD, which contains the Loop (L1)-sheet-helix (LSH) motif, two large loops L2 and L3, the direct DNA contact areas including the zinc-binding site, and the hotspots [5, 9, 51, 52]. Few previous studies that report *TP53* mutation status in PCNSL reveal incidences <10 % [10, 31], while, paradoxically, other investigations show higher incidences of p53 protein expression by immunhistochemistry (IHC) (29–60 %) as a surrogate marker of *MUT-TP53* [3, 6].

The members of the miR34-family (A/B/C) have been recognized as tumor suppressors, which are implicated in a variety of cellular processes that control carcinogenesis in concert with p53 [13, 19]. The miR34s have been placed at the center of the cell-cycle and apoptosis regulation, and loss of miR34A has been associated with poor response to therapy [4, 20, 25]. We recently investigated the *MIR34-*family in a cohort of 150 primary DLBCL patients, and identified promoter methylation, and hence inactivation, of *MIR34A* in 28 % of the patients, and thereby showed that concomitant (”double hit”) *TP53* mutation and *MIR34A* methylation were related to an exceedingly poor median survival of only 9.4 months [4, 20]. We also investigated the methylation status of DAPK (death-associated protein kinase), another player in the p53 signaling network in DLBCL, and showed that both overall- and allele specific DNA methylation correlate with poor outcome. Here, patients with concomitant *TP53* mutation also had a tendency of poorer survival [22].

Since these studies suggest that genetic- and epigenetic disruption of the p53-miR34A-DAPK network may be associated with worse outcome in DLBCL patients, we speculated whether this pathway was also disrupted in CNS DLBCL. Thus, the purpose of the current study was to investigate and characterize the structural subsets of *TP53* mutations, and to evaluate these data in the context of the *MIR34* family- and *DAPK* promoter methylation status to the clinical outcome in a large cohort of patients with PCNSL of the DLBCL subtype.

## Methods and materials

### Study population and patient samples

All patients, consecutively diagnosed with PCNSL of the DLBCL subtype at our institution (University Hospital of Copenhagen, Rigshospitalet) during a period of 13 years (2001–2014), were included. A total of 107 histological specimens, which were eligible for further histopathological examination and DNA extraction for molecular analyses, were reclassified by the authors HDMP, HB, and ER (one training- and two specialist neuro- and hematopathologists) according to the WHO-classification [44]. In addition, data were extracted and critically reviewed from the patient files, the national Danish Pathology Database (Patobank), and the national Danish Lymphoma Database (LYFO).

The clinical data included date of diagnosis, age, sex, WHO performance status, serum lactate dehydrogenase (LDH), International Prognostic Index (IPI), 1st line treatment by chemo- and immunotherapy, whole brain radiotherapy (WBRT) including number of fractions and dose, adjuvant WBRT, response to treatment, relapse, and death or last follow-up date.

### Immunohistochemistry

Assessment of protein expression by immunohistochemistry (IHC) was done by re-evaluating a panel of antibodies against: CD3, CD5, CD20, CD79A, CD10, BCL2, BCL6, MUM1, and MIB-1. A general cut-off level of 30 % was applied for all, except BCL2, where the cut-off was set at 70 % [16]. p53 protein expression was assessed by immunohistochemistry (IHC), see Additional file 1: Methods 1, and evaluated semiquantitatively. On-slide controls were used. No expression was defined as 0–9 %, and presence of expression was evaluated in intervals of 10–25 %, 25–50 %, 50–75 %, 75–100 %. In the survival analysis cut-off thresholds above 10 % were applied, as described by others [52, 53]. For the proliferation factor MIB-1 the percentage of stained nuclei was evaluated.

### DNA extraction from FFPE tissue

From all samples, DNA was isolated from 4 to 8 10 μm FFPE (formalin-fixed paraffin-embedded) tissue sections using the RecoverAll^TM^ Total Nucleic Acid Isolation Kit (Life Technologies, Carlsbad, CA, USA) according to the manufacturers’ guidelines. Paraffin was removed from the samples by Xylene incubation at 50 °C followed by ethanol wash. Proteins were degraded by digestion buffer and Proteinase-K digestion at 50°/80 °C. Subsequent isolation of DNA was performed by addition of isolation buffer and ethanol wash, followed by binding to a spin-column system, and RNA degradation by RNAse treatment. Total DNA was eluted in a 60 μL elution volume. The quantity (260 nm) and quality (260/280 ratio) of total DNA was measured by spectrophotometry on a NanoDrop-1000 spectrophotometer (Thermo Scientific, Delaware, USA).

### Detection and structural classification of *TP53* mutations

The coding sequences and splice sites of exons 5–8 of the *TP53* gene were screened for mutations by PCR and denaturing gradient gel electrophoresis (DGGE) as described previously [4, 17, 52]. By covering exon 5–8 it is expected that ~95 % of *TP53* mutations will be detected [51, 52]. Positive samples were subjected to Sanger sequencing [51]. All mutations were confirmed twice from DNA of the original sample.

Structural classification and nomenclature were applied using the IARC-TP53-Database (www.p53.iarc.fr) version R17 [35]. The types of *TP53* mutation (missense, nonsense, deletion, insertion) and the sites of mutation (exon, codon, functional domain, conserved area, CpG-site relation, functional transactivation activity, and SIFT-class) were determined. For tumors with more than one mutation, the data for each mutation were recorded as separate entries in the absolute number of mutations, but analyzed as single events for survival. The most deleterious, i.e. functionally inactivating, mutations were selected as representative for survival analysis in these double mutated cases as in previously published studies [52]. Single nucleotide variations (SNVs) were scanned in the IARC-TP53-Database for detection of validated single nucleotide polymorphisms (SNPs) [35, 47], and excluded. As described thoroughly by Young et al. [52], we compared the survival of patients with *MUT-TP53* within mutational subgroups or with the survival of *WT-TP53* patients. Structural subsets, which were applied as factors in the survival analysis, included *TP53* mutations located at hotspots in the DBD including codons involved in direct DNA contact, at the zinc-binding site, as well as frequent destabilizing mutations in β-sheets. In the analysis, *TP53* mutations located in Loop-L2, Loop-L3, and LSH-motifs, conserved areas 2–5, and CpG-sites were also incorporated, as well as functional properties including p53 activity and SIFT-class, for details, see Additional file 1: Methods 2.

### Bisulfite conversion

For initial bisulfite conversion, 250 ng (*MIR34A/B/C*) and 125 ng (*DAPK*) of DNA/sample were applied using the EZ DNA Methylation Kit (Zymo Research) according to the manufacturers’ recommendations.

### Methylation-specific melting curve analysis of *MIR34A/B/C*

The methylation status of the *MIR34A* and *MIR34 B/C* promoters was examined using methylation-specific melting curve analysis (MS-MCA); The amplification was carried out on a LightCycler® 480 instrument II (Roche Diagnostics) [4, 18, 24, 49]. The DNA sequences analyzed for promoter hypermethylation are located near the transcription start sites and in the promoter CpG-islands as previously described [4, 7, 24, 46]. The melting peaks were calculated using the LightCycler® 480 Software, Release 1.5.0SP3. Methylated DNA (Chemicon, Millipore, Billerica, MA, USA), unmethylated DNA (Qiagen), and a no template control (NTC) were included in all experiments.

### *DAPK* methylation analysis using allelic MSP-pyrosequencing

The *DAPK* methylation specific PCR (MSP) primers target the antisense strand and amplify the region surrounding the rs13300553 SNP (A/G), as described previously [23, 24]. For primer sequences and detailed methods see [22, 23], and Additional file 1: Methods 3.

### Statistics

The statistical software used for the analyses was SPSS version 22 for Windows (SPSS Inc. IBM, Armonk, NY, USA) and R version 3.2.1 for Mac OS X, Leopard (Apple, Cupertino, CA, USA). Analysis of categorical data and their in between associations was done by Fisher’s exact test, whereas t-test was used for continuous data. Survival was calculated based on survival status 5 years (60 months) after date of diagnosis. Survival analyses were carried out using the Kaplan-Meier method, and the estimates were evaluated by the log-rank test. The main outcomes were median overall survival (OS) and progression free survival (PFS). Cox proportional hazard method was applied to evaluate which variables were independent prognostic factors. The co-factors of the fitted models (sex, age, IPI) were tested for proportionality using the Cox-Aalen test. The significance level was defined as *P*-values < 0.05. All *P*-values were two-sided.

### Ethics, consent and permissions

The project was conducted according to the Declaration of Helsinki, and was approved by the regional ethics committee (H-3-2012-127) and by the Danish data protection committee (2007-58-0015) according to the Danish ethical regulations.

## Results

### Clinical and survival parameters in 107 PCNSL patients

*TP53* mutation analysis was successful in 86/107 patients, and promoter methylation studies of the *MIR34A, MIR34B/C*, and *DAPK* were successful in 93/107, 84/107 and 75/107 patients, respectively. The clinicopathological data of the whole cohort (*N* = 107) and the *TP53, MIR34A,* and *DAPK* subgroups are listed in Additional file 2: Table S1a-d. These tables also include description of the different treatment regimens and relevant doses that patients received as 1st line therapy.

The median OS for the whole cohort was 9.2 months [CI95%: 4.0–14.4], and the PFS was 6.7 months [CI95%: 3.7–9.6]. The mean and median follow-up time were 22.4 and 9.1 months (range: 0.1–157.5 months), respectively.

**Fig. 1:**
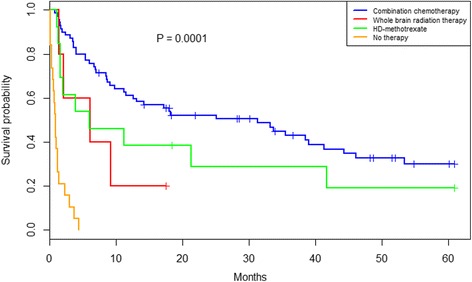
Overall survival associated with 1st line treatment in 107 PCNSL patients. Kaplan-Meier plot of 107 PCNSL patients, who were treated with combination chemotherapy +/− rituximab, whole brain radiation therapy (WBRT), high-dose (HD) methotrexate, and no therapy, *P <* 0.0001

Survival differed significantly according to 1st line treatment modality. The 70 patients that received combination chemotherapy with or without rituximab (CCT-treated +/− rituximab) had a significantly longer median OS of 31.3 months [CI 95 %: 12.6–44.3], as compared to patients receiving monotherapy with high-dose methotrexate (HD-MTX), 6.0 months [CI 95 %: 1.6-NA], whole brain radiation therapy (WBRT), 6.0 [CI 95 %: 2.1-NA], or no therapy, 0.83 months [CI 95 %: 0.57–2.2]*, P* < 0.0001, Fig. 1.

Disease status according to 1st line treatment is recorded in Additional file 2: Table S2a. At the end of follow-up, 23 (21.5 %) patients were alive, and 20/23 in partial- or complete remission; 84 (78.5 %) patients were deceased. Twenty-two patients suffered a relapse after 1st line treatment (19 CCT +/− rituximab, and 3 HD-MTX only), and received a second treatment, Additional file 2: Table S2a-b. The median time to relapse after 1st treatment in this group was 8.4 months (range: 1–60.8). Among these patients, 2 (9.1 %) patients were in complete remission and alive 51.5 and 52.0 months after diagnosis, respectively, the remaining 20 (90.9 %) had deceased. The two surviving patients were originally treated with CCT + rituximab.

### Histopathology and immunohistochemistry

**Fig. 2:**
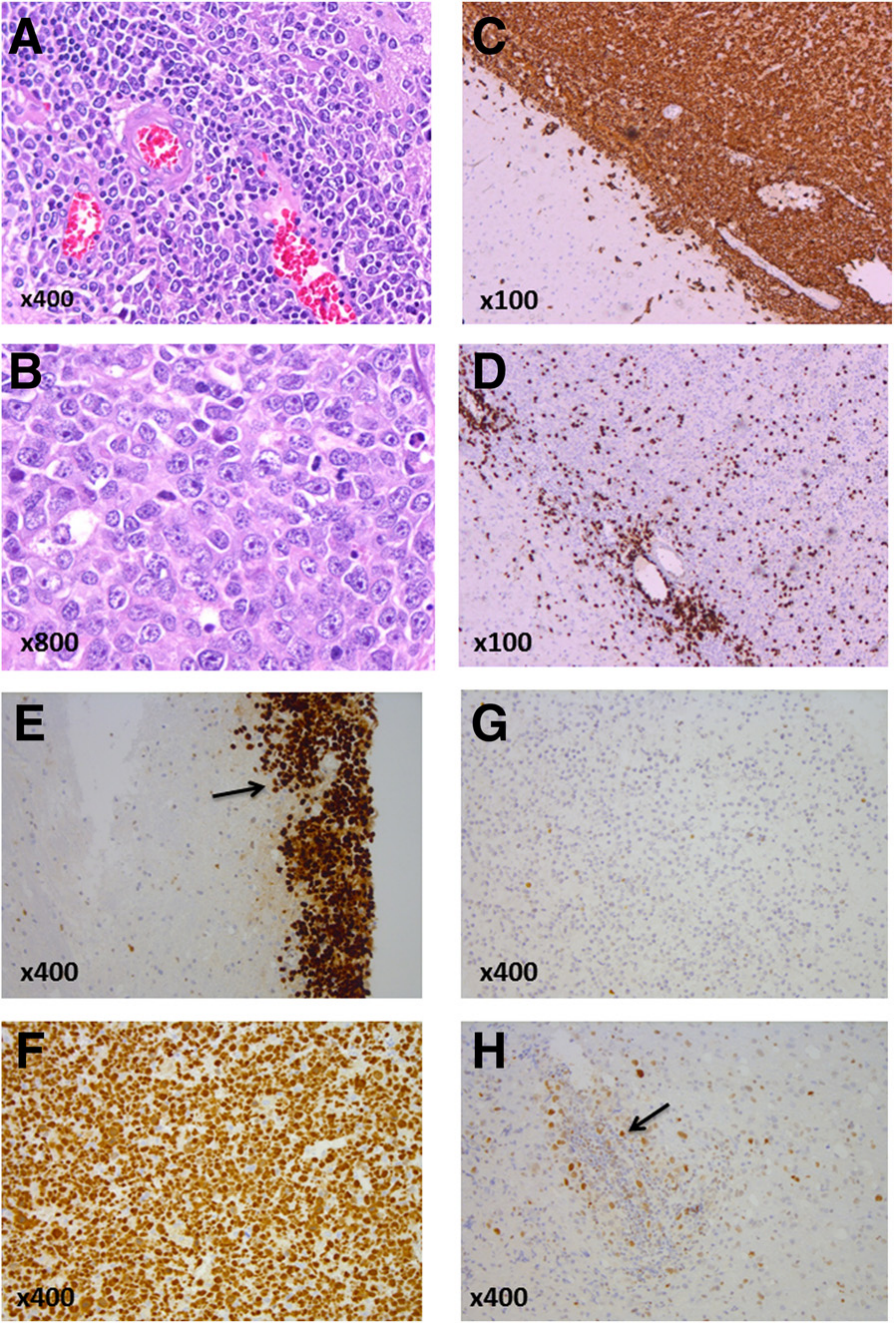
Histology and immunohistochemistry of PCNSL of the DLBCL-subtype. PCNSL composed of large lymphocytic cells with irregular immunoblastic and centroblastic nuclei, increased number of mitoses, and (**a**) perivascular-, or (**b**) diffuse lymphoma infiltration. Immunohistochemical (IHC) marker expression includes (**c**) CD20+ (B-cell staining) and (**d**) CD3- (B-cells not staining, reactive T-cells+). IHC staining pattern of p53 protein in PCNSL tissue samples: (**e**) p53++ (black arrow) in patient ID18 with a hotspot/direct DNA contact *MUT-TP53*, p.R175H, (**f**) p53++ in patient ID14 with a hotspot/direct DNA contact *MUT-TP53*, p.R248Q. (**g**) p53-, in a patient with *WT-TP53*, and (**h**) p53+, in a patient with *WT-TP53*. Perivascular staining of p53 and neoplastic B-cells (black arrow)

HE-staining showed classical patterns of CNS DLBCL with malignant angiocentric lymphocytic infiltration or diffuse sheets of B-type lymphoma (CD20+, CD79+, CD3-, CD5-) with immuno- or centroblast morphology, Fig. 2a-d.

IHC marker expression of the whole cohort and the three subgroups are shown in Additional file 2: Table S3 a-d. Briefly, the PCNSLs had an IHC-profile with the majority expressing BCL2+ (62.3 %), BCL6+ (74.6 %), CD10- (81.7 %), MUM1+ (96.7 %). Staining intensity of p53-IHC varied within specimens, and the number of nuclei expressing p53 protein was highly variable, suggesting tumor heterogeneity (Fig. 2e-h). In 86 % of the specimens p53-IHC positivity was present.

### Structural mutation profile of the *TP53* gene in PCNSL patients

Thirty-two of 86 (37.2 %) patients had one or more *TP53* mutations, resulting in 37 missense mutations, one deletion, and one nonsense mutation. Thus, 7 (8 %) patients carried two mutations. Two SNVs occurred three times each; p.R175H (hotspot) and p.M169I, and p.T140I twice, leaving 34 different SNVs detected. The classification of the absolute number of mutations is shown in detail in Table 1, which is divided in two; the upper part (patient ID’s 1–24) representing patients that received CCT-treatment, and the lower part (patient ID’s 25–32) representing patients that received other treatment, but no CCT-therapy.

**Table 1:** Structural classification of *TP53* mutations in primary central nervous system lymphoma. The structural classification includes an absolute number of 39 *TP53* mutations divided on 32 patients, of which 24 combination chemotherapy treated patients harbored 28 mutations (IDs 1–24), and the remaining 8, who harbored 11 mutations, received other therapies (IDs 25–32)

ID	Exon	*TP53* Mutation	Sequence change	Protein change	Basepair change	Localization	Hotspot mutation	Direct DNA contact/Zn	CpG Site	Conserved region	Siftclass	Mutation type	Transactivation class
1	5I	CYS-135-TYR	TGC > TAC	p.C135Y	C.404G > A	LSH				2	Deleterious	Missense	Non-Active
2	5I	THR-140-ILE	ACC > ATC	p.T140I	C.419C > T	β				2	Deleterious	Missense	Partially Active
3	5I	PRO-142-SER	CCT > TCT	p.P142S	C.424C > T	β				2	Deleterious	Missense	Partially Active
4a	5I	GLN-144-STOP	CAG > CTG	p.Q144	C.430C > T	β				99	Deleterious	Nonsense	NA
5a	5I	ASP-148-ASN	GAT > AAT	p.D148N	C.442G > A	L2				3	Neutral	Missense	Active
6	5II	MET-169-ILE	ATG > ATA	p.M169I	C.507G > A	L2				3	Partially Deleterious	Missense	Partially Active
7	5II	MET-169-ILE	ATG > ATA	p.M169I	C.507G > A	L2				3	Partially Deleterious	Missense	Partially Active
8	5II	MET-169-ILE	ATG > ATA	p.M169I	C.507G > A	L2				3	Partially Deleterious	Missense	Partially Active
9	5II	GLU-171-LYS	GAG > AAG	p.E171K	C.511G > A	L2				3	Deleterious	Missense	Active
10a	5II	VAL-173-MET	GTG > ATG	p.V173M	C.517G > A	L2				3	Deleterious	Missense	Non-Active
10b	5II	ARG-181-HIS	CGC > CAC	p.R181H	c.542G > A	L2			CpG	3	Deleterious	Missense	Partially Active
11	5II	ARG-175-HIS	CGC > CAC	p.R175H	C.524G > A	L2	Hotspot	Direct DNA Contact	CpG	3	Deleterious	Missense	Non-Active
12	5II	ARG-175-HIS	CGC > CAC	p.R175H	C.524G > A	L2	Hotspot	Direct DNA Contact	CpG	3	Deleterious	Missense	Non-Active
5b	5II	Frameshift	deletion	NA	C.528.del1	L2		Zn-binding Site	CpG	3	Frameshift	Deletion	NA (Zn-binding Site)
13	5II	HIS-179-ARG	CAT > CGT	p.H179R	C.536A > G	L2	Hotspot	Zn-binding Site		3	Deleterious	Missense	Non-Active
14	5II	ASP-184-GLY	GAT > GGT	p.D184G	C.551A > G	L2				3	Neutral	Missense	Active
15	6	ARG-202-CYS	CGT > TGT	p.R202C	C.604C > T	β			CpG	99	Deleterious	Missense	Active
16a	6	ASP-208-GLY	GAC > GGC	p.D208G	C.623A > G	β				99	Deleterious	Missense	Active
17	7	TYR-236-CYS	TAC > TGC	p.Y236C	C.707A > G	β				99	Deleterious	Missense	Non-Active
4b	7	MET-237-VAL	ATG > GTG	p.M237V	C.709A > G	L3				99	Deleterious	Missense	Non-Active
18	7	ASN-239-SER	AAC > AGC	p.N239S	C.716A > G	L3	Hotspot	Direct DNA Contact		4	Deleterious	Missense	Non-Active
19	7	ARG-248-GLN	CGG > CAG	p.R248Q	C.743G > A	L3	Hotspot	Direct DNA Contact	CpG	4	Deleterious	Missense	Non-Active
20	7	ARG-249-LYS	AGG > AAG	p.R249K	C.746G > A	L3	Hotspot	Direct DNA Contact		4	Deleterious	Missense	Non-Active
16b	7	THR-256-ILE	ACA > ATA	p.T256I	C.767C > T	L3				4	Deleterious	Missense	Partially Active
21	8	ARG-273-CYS	CGT > TGT	p.R273C	C.817C > T	LSH	Hotspot	Direct DNA Contact	CpG	5	Deleterious	Missense	Non-Active
22	8	GLY-279-GLU	GGG > GAG	p.G279E	C.836G > A	LSH				5	Deleterious	Missense	Non-Active
23	8	ARG-280-LYS	AGA > AAA	p.R280K	C.839G > A	LSH	Hotspot	Direct DNA Contact		5	Deleterious	Missense	Non-Active
24	8	GLU-286-LYS	GAA > AAA	p.E286K	C.856G > A	LSH				5	Deleterious	Missense	Non-Active
25a	5I	THR-140-ILE	ACC > ATC	p.T140I	C.419C > T	β				99	Deleterious	Missense	Partially Active
26a	5I	PRO-151-SER	CCC > CCT	p.P151S	C.451C > T	β		Direct DNA Contact		99	Deleterious	Missense	Non-Active
27	5I	ILE-162-MET	ATC > ATG	p.I162M	C.486C > G	β				99	Deleterious	Missense	Partially Active
28a	5II	VAL-172-ALA	GTG > GCG	p.V172A	C.515 T > C	L2				3	Deleterious	Missense	Active
29	5II	ARG-175-HIS	CGC > CAC	p.R175H	C.524G > A	L2	Hotspot	Direct DNA Contact	CpG	3	Deleterious	Missense	Non-Active
30	5II	GLY-187-ASP	GGT > GAT	p.G187D	C.560G > A	L2				3	Neutral	Missense	Active
26b	5II	GLY-187-ASP	GGT > GAT	p.G187D	C.560G > A	L2				3	Neutral	Missense	Active
31	7	MET-243-ILE	ATG > ATA	p.M243I	C.729G > A	L3		Direct DNA Contact		4	Deleterious	Missense	Non-Active
28b	8	VAL-272-MET	GTG > ATG	p.V272M	C.814G > A	LSH				5	Deleterious	Missense	Non-Active
32	8	ARG-290-CYS	CGC > TGC	p.R290C	C.868C > T	β			CpG	99	Deleterious	Missense	Active
25b	8	GLY-302-ARG	GGG > AGG	p.G302R	C.904G > A	C-Term				99	Neutral	Missense	Active

The structural analysis was restricted to the 24 CCT-treated patients in concordance with the survival analyses described below. The distribution pattern of these 28 SNVs (4 patients had two *TP53* mutations) was analyzed according to their position in the 3-dimensional crystal model; 22/28 (78.6 %) were localized in codons involved in DNA-binding motifs of the central core domain. These included 5 (17.9 %) in the Loop-L3 (codons 237–250) that interacts with the minor groove of the DNA, and 5 (17.9 %) in the LSH-motif (codons 119–135 and 272–287) that interacts with the major groove of the DNA, and 12 (42.9 %) in the Loop-L2 (codons 164–194), which enhances the binding affinity between the DNA-helix and *TP53* under physiological conditions. In 9 (32.1 %) of the absolute numbers of mutations, the mutated amino acid residues were involved in areas with hotspot properties, direct DNA contact or the central Zn-binding site, Table 1. All hotspot mutations were integrated in the direct DNA contact group. Location of *MUT-TP53* according to exons, highly conserved areas and the distribution of basepair shifts are illustrated in Additional file 3: Figure S1a-c. The missense mutations could be classified according to their functionality, revealing the capacity of transactivating the promoter regions of several p53 target genes. Among the missense mutations, 14 (50.0 %) were predicted to inactivate p53, 7 (25.0 %) were associated with partially active p53, and 5 (17.9 %) with active p53. Among the remaining 2 (7.1 %), a single base deletion was allocated to the Zn-binding site. The SIFT-class prediction model designated 22 (78.6 %) of the *TP53* mutations as being deleterious, Table 1.

### Association between *TP53* mutations, clinical characteristics and survival

In the 86 patients with a successful *TP53* mutational analysis, complete or partial clinical data was available, Additional file 2: Table S2b. There were no significant differences in the clinical presentation patterns between the 32 patients that carried one or more *TP53* mutations compared to the 54 patients with *WT-TP53.*

In these 86 patients, survival analysis did not reveal any differences in OS between PCNSL cases with *MUT-TP53* versus *WT-TP53* (Median OS: 13.1 vs. 9.1 months respectively, *P =* 0.625). At the end of follow up, 27/32 (84.4 %) of the *MUT-TP53* cases had deceased as opposed to 41/54 (75.9 %) of the *WT-TP53* cases, and no differences in OS were seen between *MUT-TP53* cases with 1 or 2 mutations, respectively (*P =* 0.46).

**Fig. 3:**
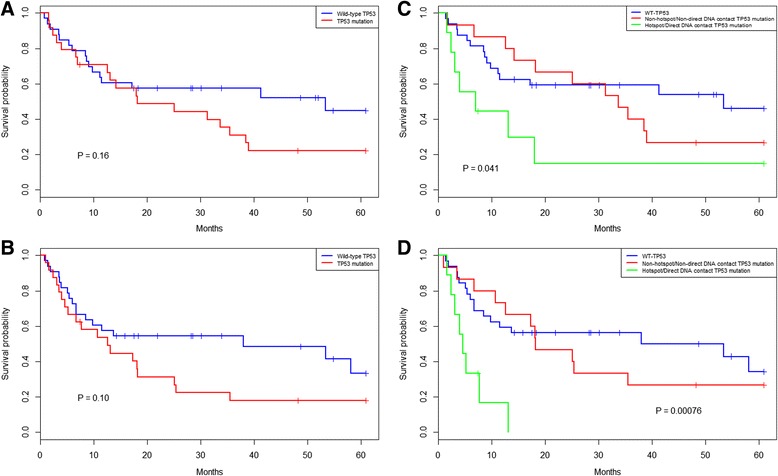
Overall- and progression free survival of 57 PCNSL CCT-treated patients subjected to *TP53* mutation analysis. Patients with *MUT-TP53* (red) (*n* = 24) showed a non-significant trend towards worse (**a**) OS, and (**b**) PFS compared to patients with *WT-TP53* (blue)*, P =* 0.16 and *P* = 0.10, respectively. Presence of a *TP53* hotspot/or direct DNA contact mutations (*n* = 9) was indicative of poorer (**c**) OS, and (**d**) PFS compared to patients with non-hotspot/non-direct DNA contact *TP53* mutations and *WT-TP53*, *P =* 0.041 and *P =* 0.00076, respectively

When the analyses were confined to the 57/86 (65.5 %) CCT-treated patients, a potential biological difference between PCNSL harboring *MUT-TP53* versus *WT-TP53* was observed, however, this was non-significant, Fig. 3a, b. This observation prompted us to perform survival analyses based on the predicted structural subsets in the group of CCT-treated PCNSL patients.

### Prognostic impact of *TP53* hotspot mutations/direct DNA contact in CCT-treated patients

Because all hotspot mutations (*n* = 8) were an integrated part of the direct DNA contact group (*n* = 9), we chose to merge these groups into one. When the survival analyses were confined to the cohort of the 57 CCT-treated PCNSL, the OS and PFS differed significantly for patients with hotspot/direct DNA contact- (*n* = 9) vs. non-hotspot/non-direct DNA contact *MUT-TP53* (*n* = 15) vs*. WT-TP53* (*n* = 32); in the OS analysis the median was 6.9 vs 33.7 vs 53.4 months, respectively, *P =* 0.041 whereas for the PFS, the median was 4.6 months vs. 18.2 vs 45.7 months*, P =* 0.00076, Fig. 3c, d. In an univariate- and multivariate Cox regression model, presence of a hotspot/direct DNA contact *MUT-TP53* was highly predictive for poor outcome in both univariate- and multivariate analyses for both PFS *P* = 0.0046 (HR: 1.97, [CI95%: 1.97–3.01]) and *P =* 0.0083 (HR: 1.95, [CI95%: 1.19–3.21]), respectively, and OS *P* = 0.028 (HR: 1.67, [CI95%: 1.06–2.67]) and *P* = 0.012 (HR: 1.86, [CI95%: 1.14–3.01]), respectively, Table 2.

**Table 2:** Univariate- and multivariate Cox proportional hazard regression models of analyzed factors and clinical co-factors. Significant *P*-values are written in italics

Survival outcome	Univariate survival analysis	Multivariate survival analysis
Factor	Cox proportional hazard model	Cox proportional hazard model
Co-factors	*P*-value (Hazard ratio, [95 % confidence intervals])	*P*-value (Hazard ratio, [95 % confidence intervals])
Progression free survival		
Hot Spot/Direct DNA Contact *MUT*-*TP53*	*0.0046* (1.97, [1.14-3.01])	*0.0083* (1.95, [1.19–3.21])
Age		0.94 (1.0, [0.96–1.05])
Sex		
Female		1.00 (1.00, [1.00–1.00])
Male		0.18 (0.63, [0.32–1.25])
IPI		
Low		1.00 (1.00, [1.00–1.00])
High		0.36 (1.45, [0.65–3.24])
Overall survival		
Hot Spot/Direct DNA Contact *MUT*-*TP53*	*0.028* (1.67, [1.06–2.67])	*0.012* (1.86, [1.14–3.01])
Age		0.20 (1.03, [0.98–1.08])
Sex		
Female		1.00 (1.00, [1.00–1.00])
Male		0.35 (0.71, [0.34–1.46])
IPI		
Low		1.00 (1.00, [1.00–1.00])
High		0.42 (1.44, [0.59–3.48])
Progression free survival		
Double Hit, *MUT-TP53* and *MIR34A*-meth	*0.010* (2.96, [1.29–6.76])	0.066 (2.30, [0.95–5.57])
Age		0.73 (0.99, [0.95–1.04])
Sex		
Female		1.00 (1.00, [1.00–1.00])
Male		0.07 (0.50, [0.24–1.06])
IPI		
Low		1.00 (1.00, [1.00–1.00])
High		0.26 (1.69, [0.68–4.19])

*MUT-TP53* located to Loop-L2, Loop-L3, and LSH-motifs, as well as mutations in the conserved regions 2–5, or according to functional properties did not have any association with survival outcomes (data not shown).

### *MIR34A* and *MIR34B/C* methylation status and survival outcome

Promoter methylation status of *MIR34A* and *MIR34B/C* was successfully investigated in 93 and 84 patients. The clinical data of patients with or without methylation of *MIR34A* are shown in Additional file 2: Table S2c. Fifty-three of 93 (57.0 %) patients had promoter methylation of *MIR34A,* and 80/84 (95.2 %) patients had promoter methylation of *MIR34B/C.* No survival analysis was carried out for *MIR34B/C*, since only 2 events occurred among the 4 patients designated as un-methylated.

No association between PFS or OS and promoter methylation of *MIR34A* alone was detected, neither in the entire cohort, *P =* 0.63 and *P =* 0.84, respectively, nor in the CCT-treated subcohort, *P =* 0.58 and *P =* 0.19, respectively, Additional file 3: Figure S2a, b.

### Impact of concomitant *TP53* mutation and *MIR34A* methylation on survival

**Fig. 4:**
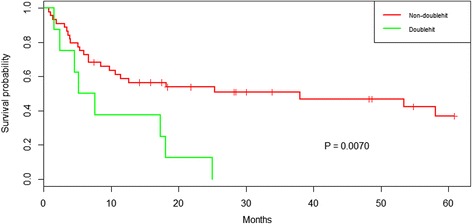
Concomitant *TP53* mutation and *MIR34A* methylation “double-hit” associated with poorer progression free survival in CCT-treated PCNSL patients. Kaplan-Meier plot of 52/57 PCNSL patients, of which the 8 patients with “double-hit” *TP53* mutation and *MIR34A* promoter methylation had a significantly poorer PFS*. P =* 0.0070

Seventy-eight PCNSL cases, which had been analyzed for both *MUT-TP53* and *MIR34A* promoter methylation, were matched pairwise to detect presence of “double hit”. Eleven of 78 (14.1 %) had “double hit”. The remaining 67/78 patients (85.9 %) that either had a *TP53* mutation or a *MIR34A* promoter methylation, i.e. “single hit”, or neither of them, were grouped together as “non-double hit”. In the CCT-treated subcohort, 52/54 (96.3 %) of the patients entered the analysis, of whom 8/52 (15.4 %) had “double hit”. Presence of a “double hit” was associated with a poorer PFS in the CCT-treated subgroup, as compared to “non-double hit”. Eight of 8 (100 %) CCT-patients with “double hit” had all experienced an event at follow-up (median PFS 6.4 months), in comparison to 24/44 (54.5 %) in the “non-double hit” subgroup, (median PFS 38.0 months), *P* = 0.0070, Fig. 4.

Noteworthy, among the CCT-treated patients 5/8 of the “double hit” cases were hotspot/direct DNA contact mutations (ID’s 5, 11, 20, 21, 23), and 3 non-hotspot/non-direct DNA contact mutations (ID’s: 2, 7, 8). In the Cox regression model, presence of a “double-hit” was significant only in the univariate-, but not in the multivariate model, *P* = 0.010 (HR: 2.96, [CI95%: 1.29–6.76] and P = 0.066 (HR: 2.30, [CI95%: 0.95–5.57]), Table 2.

### *DAPK* methylation status and survival

Seventy-five of 107 (70.1 %) PCNSL FFPE samples were successfully tested for *DAPK* promoter methylation and 70/75 (93.3 %) were methylated. The clinical data of patients with or without methylation of *DAPK* are presented in Additional file 2: Table S2d. There was no significant difference between OS/PFS of patients with or without methylation of *DAPK* in the entire cohort, when analyzed in CCT-treated patients only, or when combined with *MUT-TP53* data (data not shown). Data on the rs13300553 SNP genotypes and allelic *DAPK* methylation patterns are presented in Additional file 4: Data.

## Discussion

Here, we present data on a large cohort of 107 consecutively diagnosed PCNSL cases, with comprehensive analyses of clinical data, histopathology, *TP53* mutation status, and *MIR34A/B/C* and *DAPK* promoter methylation. At the time of diagnosis 80 of 107 (75 %) cases had disruption of one or more of the investigated genes, indicating that disrupted p53-signaling may be an essential part of PCNSL pathogenesis, which may contribute to the aggressive phenotype.

We did not observe a general negative effect on outcome by *TP53* mutations in our entire cohort. However, when analyzing the subcohort of CCT-treated PCNSL patients, Kaplan-Meier analysis showed an inverse relationship with both PFS and OS in cases with *MUT-TP53* in hotspots/direct DNA contact residues, compared to cases with *WT-TP53* or other types of *MUT-TP53*. This observation was emphasized in multivariate Cox regression analyses, which showed that presence of a hotspot/direct DNA contact mutation was an independent negative prognostic factor for survival in CCT-treated PCNSL patients when adjusting for sex, age and IPI-status.

Previous studies have shown that *TP53* mutation status plays a pivotal role in predicting outcome in patients with peripheral DLBCL treated with R-CHOP/CHOP-like chemotherapy: Patients with *MUT-TP53* have a 2-fold higher relative risk of death compared to patients with *WT-TP53*, and patients with *TP53* mutations that involved hotspot residues, and overlapping direct DNA contact residues of the DBD had a high mortality rate [51–53]. Our study of PCNSL consolidates the importance of not only reporting the mere *MUT-TP53* number, but combines the structural classification in the survival analyses, stressing the inverse association to outcome when PCNSL patients harbor hotspot/direct DNA contact *MUT-TP53*, as formerly shown in DLBCL and other cancer subtypes [12, 26, 35, 42].

The reported frequency of somatic *TP53* mutations in lymphomas varies significantly [50], even within individual lymphoma subtypes across different studies, which is likely due to differences in the quality of study material and the applied methods [26]. *TP53* mutation status in PCNSL has only sporadically been reported in two other cohorts revealing much lower incidences compared to our study [10, 31]. In Cobbers’ pioneering work, 20 PCNSL patient samples (11 freshly frozen and 9 FFPE) were subjected to single-strand conformation polymorphism analysis followed by gel electrophoresis and DNA sequencing of *TP53* exons 5–8, discovering only one missense mutation at codon 248 in one of twenty (5 %) patients [10]. In a recent work, 71 PCNSL patient samples (48 freshly frozen and 23 FFPE) were screened for mutations in 21 genes using the Ion Torrent NGS-platform, revealing a *TP53* mutation rate of 7 %. However, validation by alternative methods was not performed for *TP53*, and no detailed structural classification of *MUT-TP53* was provided in this study [31]. The primers of the current commercial Ion Torrent NGS-platform would a priori only cover 70.6 % of the detected *TP53* mutations in our study; some genomic areas may be difficult to assess (e.g. homopolymeric tracts), and intra- and intertumor heterogeneity may require additional depth and coverage [28, 38].

When considering the reported low frequency of *MUT-TP53* in PCNSL, we speculated whether the observed high frequency could be influenced by sequencing artifacts resulting from the use of FFPE DNA [2], or if it represented a genuine high frequency of driving mutations with clinical impact accompanied by random passenger mutations of unknown relevance [8, 12, 41, 45]. The fact that up to one mutation artifacts per 500 bases have been reported in methodological analyses of FFPE tissue in DNA sequencing with the majority of transitions being of C > T or G > A type [48] could be an indication of the former situation [12]. However, the previous studies mentioned above also included a significant proportion of FFPE samples in their cohorts, and we have not observed notable higher frequencies of *TP53* mutations in FFPE compared to fresh frozen samples in peripheral DLBCL using the same methodology [22]. Finally, it could be argued that we would not observe the clear inverse relationship between hotspot/direct DNA contact *TP53* mutations and clinical survival parameters, confirmed by multivariate Cox regression analysis, if a significant proportion of the detected mutations were randomly occurring artifacts. Thus, taken together, our data suggest that the frequency of *TP53* mutations in PCNSL is higher than what has previously been appreciated and consolidate the prognostic impact of hotspot/direct DNA contact *MUT-TP53* in PCNSL.

Interestingly, we also observed higher frequencies of *MIR34A/B/C* and *DAPK* promoter methylation than we previously detected in DLBCL outside the CNS [4, 22]. As in extra-CNS DLBCL we identified cases with concomitant *MUT-TP53* and *MIR34A* methylation to be associated with a poor PFS. When combined with *MIR34A* methylation, also *MUT-TP53* outside the hotspot/direct DNA contact loci seem to influence outcome emphasizing the synergistic function of these aberrations in lymphomagenesis. However, although we provide one of the largest molecular studies of PCNSL to date, the numbers of cases in the individual groups are still small. Ideally, the prognostic impact of hotspot/direct DNA contact *TP53* mutations and concomitant *MUT-TP53* and *MIR34A* methylation observed in this study, should be confirmed in other independent cohorts. NGS is a preferred methodology in future *MUT-TP53* investigations, however, at least initially; the observed mutational spectrum should be compared to that obtained by other standard methods.

## Conclusion

In conclusion, our data indicate that PCNSL have comprehensive molecular alterations in the p53-pathway, and that the high disruption frequency of the p53-pathway contributes to the poor outcome in CCT-treated PCNSL patients. In a clinical perspective, *TP53* mutational and *MIR34A* methylation analyses of PCNSL could serve as additional tools in the prognostic setting when choosing treatment strategy. With referral to the accumulating evidence that p53 hotspot mutants have multiple gain of function properties [43], they may potentially serve as therapeutic targets. Thus it is tempting to speculate whether disruption of the p53-pathway may identify patients that are candidates for emerging, alternative treatment options in PCNSL.

## Additional files

Additional file 1:
**Methods 1**. Immunohistochemistry. **Methods 2**. Detection and structural classification of *TP53* mutations. **Methods 3**. DAPK methylation analysis using allellic MSP-pyrosequencing. (PDF 379 kb) Additional file 2: Tables S1a-d. Clinical data in 107 PCNSL patients and *TP53*, *MIR34A*, and *DAPK* subgroups. **Tables S2a-b.** Disease status (a), and treatment at relapse/progression (b). **Tables S3a-d.** Immunohistochemical marker expression in 107 PCNSL patients and *TP53*, *MIR34A*, and *DAPK* subgroups. (PDF 534 kb) Additional file 3: Figure S1a-c. Supplemental figures 1a-c. **Figure S2a-b**. Supplemental figures. (PDF 474 kb) Additional file 4: Data methylation status and survival. (PDF 262 kb)

## Abbreviations

CCT: combination chemotherapy (Additional Table 1a-d, footnote)
CI 95 %: confidence interval 95 %
CNS: central nervous system
CNS DLBCL: central nervous system diffuse large b-cell lymphoma (used synonymously with PCNSL)
DAPK: death-associated protein kinase
DBD: DNA binding domain
DGGE: denaturing gradient gel electrophoresis
DLBCL: diffuse large b-cell lymphoma
FFPE: formalin fixed paraffin embedded
HD-MTX: high-dose methotrexate
HR: hazard ratio
IARC: international agency for research on cancer
IHC: immunohistochemistry
IPI: international prognostic index
L2: loop-L2
L3: loop-L3
LDH: lactate dehydrogenase
LSH: Loop (L1)-sheet-helix
miR/MIR: miRNA/MIRNA
MSP: methylation specific PCR
MTX: methotrexate
MUT-TP53: TP53 mutations
NGS: next generation sequencing
OS: overall survival
PCNSL: primary central nervous system lymphoma (used synonymously with CNS DLBCL)
PCR: polymerase chain reaction
PFS: progression free survival
SNP: single nucleotide polymorphy
SNV: single nucleotide variation
WBRT: whole brain radiation therapy
WHO: world health organization
WT-TP53: wildtype TP53
