# Supplementary material for: TP53 hotspot mutations are predictive of survival in primary central nervous system lymphoma patients treated with combination chemotherapy
Source: Acta Neuropathol Commun. 2016 Apr 22;4:40. doi: 10.1186/s40478-016-0307-6 (PMC4840983; doi:10.1186/s40478-016-0307-6)
Supplement: Additional file 1: — Methods 1. Immunohistochemistry. Methods 2. Detection and structural classification of TP53 mutations. Methods 3. DAPK methylation analysis using allellic MSP-pyrosequencing. (PDF 379 kb) [file 40478_2016_307_MOESM1_ESM.pdf]

Title:

TP53 hotspot mutations are predictive of survival in primary central nervous system lymphoma patients treated with combination chemotherapy

Journal Name: Acta Neuropathologica Communications

Authors:

Helga D. Munch-Petersen, Fazila Asmar, Konstantinos Dimopoulos, Aušrinė Areškevičiūtė, Peter de Nully Brown, Mia Seremet Girkov, Anja Pedersen, Lene D. Sjö, Steffen Heegaard, Helle Broholm, Lasse S. Kristensen,Elisabeth Ralfkiaer, Kirsten Grønbæk

Corresponding author:

Kirsten Grønbæk

Professor, MD, DMSc.
Department of Hematology,

Rigshospitalet, Copenhagen University Hospital

Dept. 3733, Copenhagen Biocenter
Building 2, 3rd floor
Ole Maaløes Vej 5
2200 Copenhagen N
Denmark

Phone + 4535456086

Email: kirsten.groenbaek@regionh.dk

Additional methods 1

Staining of p53 by immunohistochemistry

The tissue specimens were stained with a monoclonal mouse anti-human p53-protein antibody (DAKO, Clone DO-7, reference number M7001, Agilent Technologies, Glostrup, Denmark) and run on a Ventana Benchmark Ultra (Roche, Basel, Switzerland). The applied procedures were according to the manufacturers' instruction, and the following staining protocol was used.

Protocol Summary

Procedure: U ultraView DAB (v1.02.0018)

BenchMark ULTRA IHC/ISH Staining Module

Rigshospitalet, Frederik d. V's vej 11 DK-2100, Koebenhavn. Danmark

Protocol No	Protocol Name	Creation Date
366	P53	13-11-2014

1 Deparaffinization [Selected]
2 Warmup Slide to [72 Deg C] from Medium Temperatures (Deparaffinization)
3 Cell Conditioning [Selected]
4 ULTRA Conditioner #1 [Selected]
5 Warmup Slide to [99 Deg C], and Incubate for 8 Minutes (Cell Conditioner #1)
6 20 minutes of ULTRA CC1 [Selected]
7 36 minutes of ULTRA CC1 [Selected]
8 52 minutes of ULTRA CC1 [Selected]
9 64 minutes of ULTRA CC1 [Selected]
10 Ab Incubation Temperatures [Selected]
11 Warmup Slide to [36 Deg C], and Incubate for 4 Minutes (Antibody)
12 Antibody [Selected]
13 Apply One Drop of [PREP KIT 11] (Antibody), Apply Coverslip, and Incubate for [0 Hr 32 Min]
14 Amplify [Selected]
15 Mouse Antibody Amp [Selected]
16 ultraWash [Selected]
17 Counterstain [Selected]
18 Apply One Drop of [HEMATOXYLIN II] (Counterstain), Apply Coverslip, and Incubate for [8 Minutes]
19 Post Counterstain [Selected]
20 Apply One Drop of [BLUING REAGENT] (Post Counterstain), Apply Coverslip, and Incubate for [8 Minutes]

Additional methods 2

As described thoroughly by Young et al [7], we compared *MUT-TP53* within subgroups or *WT-TP53* patients in the survival analysis.

I) *TP53* missense mutations altering codons that are involved in direct DNA contact (A119, K120, S121, N239, S241, M243, N247, R248, R249, R273, C275, A276, C277, R280, R282, and R283), those at hotspot codons (R175, R196, R213, G245, R248, R249, R273, and R282), and the zinc-binding site (C176, H179, C238, and C242) were recorded and integrated in survival analysis. Also, destabilizing tumorigenic mutations in the β-sheets were annotated; V143A, L145Q, P151S, V157F, I195T, Y220C, I232T, I255F, and I255F [1, 2, 7, 8].

II) Analysis was performed according to mutations positions and DBD-structures in a 3-dimensional crystal model including Loop-L2 (codons 164-194), Loop-L3 (codons 237-250), and the LSH-motif (codons 119-135 and 272-287). Survival outcomes between patients with mutations in the DBD versus mutations outside the DBD were compared [1, 2, 7].

III) Missense mutations that alter amino acids in highly conserved areas 2 (codons 117-142), 3 (codons 171-181), 4 (codons 234-258), and 5 (codons 270-286) [2, 7] were registered.

IV) Mutations were subdivided into functional classes based on yeast functional assays available in the database. The classes refer to the retained TA & TIA in the MUT-p53 gene product as a percent of the WT-p53. Three functional groups were applied: inactive p53, partially active p53, and active p53 [3, 6]. Another functional parameter was SIFT class, which is calculated using a bioinformatic model based on protein sequence homology. SIFT class divide missense mutations into deleterious or partially deleterious (i.e. which totally or partially disrupt the p53 function) as opposed to neutral (tolerable) [7].

V) Mutations situated at CpG-sites were recorded and incorporated in survival analysis.

VI) Nonmissense mutations were defined as any other mutation than missense mutations (frameshift, insertions, deletions, splice, or nonsense).

Additional methods 3

The *DAPK* methylation specific PCR (MSP) primers, target the antisense strand and amplify the region surrounding the rs13300553 SNP (A/G), as described previously [4, 5]. Several non-CpG cytosines in each of the primers select against the amplification of incompletely converted molecules. Additional CpG sites and non-CpG cytosines in between the primers serve as a control for the amplification of methylated and bisulfite converted template, respectively. We also applied the specific assay for unmethylated *DAPK1* sequences, which target the same region to verify that negative samples actually were negative because of unmethylation and not that the DNA was lost during bisulfite conversion. Genotyping the rs13300553 SNP was done using M-13 tagged *DAPK* specific PCR primers and sequenced using Sanger sequencing. The primer sequences have been published previously [5]. PCR cycling was performed on the Gene PCR System 9700 (Applied Biosystems). The cycling protocol started with one cycle of 95°C for 15 min, followed by 50 cycles of 94°C for 10 s, 60°C for 20 s, 72°C for 20 s, and one cycle of 72°C for 10 min. For the reaction mixtures the PyroMark PCR Master Mix (Qiagen) was used at a final concentration at 1X and a final Coral concentration of 1X. Final primer concentrations were 200 nM of each primer, and 25 ng of bisulfite converted DNA was used. The final reaction volume was 25 µL. Samples were sequenced on the PyroMark Q24 (Qiagen) using the PyroMark Gold Q24 reagents (Qiagen), according to the manufacturers' instructions.

References

1. Bullock AN, Henckel J, Fersht AR (2000) Quantitative analysis of residual folding and DNA binding in mutant p53 core domain: definition of mutant states for rescue in cancer therapy. Oncogene 19:1245–56. doi: 10.1038/sj.onc.1203434

2. Cho Y, Gorina S, Jeffrey PD, Pavletich NP (1994) Crystal structure of a p53 tumor suppressor-DNA complex: understanding tumorigenic mutations. Science 265:346–55.

3. Kato S, Han S-Y, Liu W, Otsuka K, Shibata H, Kanamaru R, Ishioka C (2003) Understanding the function-structure and function-mutation relationships of p53 tumor suppressor protein by high-resolution missense mutation analysis. Proc Natl Acad Sci U S A 100:8424–9. doi: 10.1073/pnas.1431692100

4. Kristensen LS, Johansen JV, Grønbæk K (2015) Allele-Specific DNA Methylation Detection by Pyrosequencing®. Methods Mol Biol 1315:271–89. doi: 10.1007/978-1-4939-2715-9_20

5. Kristensen LS, Treppendahl MB, Asmar F, Girkov MS, Nielsen HM, Kjeldsen TE, Ralfkiaer E, Hansen LL, Grønbæk K (2013) Investigation of MGMT and DAPK1 methylation patterns in diffuse large B-cell lymphoma using allelic MSP-pyrosequencing. Sci Rep 3:2789. doi: 10.1038/srep02789

6. Petitjean A, Mathe E, Kato S, Ishioka C, Tavtigian S V, Hainaut P, Olivier M (2007) Impact of mutant p53 functional properties on TP53 mutation patterns and tumor phenotype: lessons from recent developments in the IARC TP53 database. Hum Mutat 28:622–9. doi: 10.1002/humu.20495

7. Young KH, Leroy K, Møller MB, Colleoni GWB, Sánchez-Beato M, Kerbauy FR, Haioun C, Eickhoff JC, Young AH, Gaulard P, Piris MA, Oberley TD, Rehrauer WM, Kahl BS, Malter JS, Campo E, Delabie J, Gascoyne RD, Rosenwald A, Rimsza L, Huang J, Braziel RM, Jaffe ES, Wilson WH, Staudt LM, Vose JM, Chan WC, Weisenburger DD, Greiner TC (2008) Structural profiles of TP53 gene mutations predict clinical outcome in diffuse large B-cell lymphoma: an international collaborative study. Blood 112:3088–98. doi: 10.1182/blood-2008-01-129783

8. Young KH, Weisenburger DD, Dave BJ, Smith L, Sanger W, Iqbal J, Campo E, Delabie J, Gascoyne RD, Ott G, Rimsza L, Müller-Hermelink HK, Jaffe ES, Rosenwald A, Staudt LM, Chan WC, Greiner TC (2007) Mutations in the DNA-binding codons of TP53, which are associated with decreased expression of TRAILreceptor-2, predict for poor survival in diffuse large B-cell lymphoma. Blood 110:4396–405. doi: 10.1182/blood-2007-02-072082
