# Supplementary material for: TP53 hotspot mutations are predictive of survival in primary central nervous system lymphoma patients treated with combination chemotherapy
Source: Acta Neuropathol Commun. 2016 Apr 22;4:40. doi: 10.1186/s40478-016-0307-6 (PMC4840983; doi:10.1186/s40478-016-0307-6)
Supplement: Additional file 2: Tables S1a-d. — Clinical data in 107 PCNSL patients and TP53, MIR34A, and DAPK subgroups. Tables S2a-b. Disease status (a), and treatment at relapse/progression (b). Tables S3a-d. Immunohistochemical marker expression in 107 PCNSL patients and TP53, MIR34A, and DAPK subgroups. (PDF 534 kb) [file 40478_2016_307_MOESM2_ESM.pdf]

Title:

TP53 hotspot mutations are predictive of survival in primary central nervous system lymphoma patients treated with combination chemotherapy

Journal Name: Acta Neuropathologica Communications

Authors:

Helga D. Munch-Petersen, Fazila Asmar, Konstantinos Dimopoulos, Aušrinė Areškevičiūtė, Peter de Nully Brown, Mia Seremet Girkov, Anja Pedersen, Lene D. Sjö, Steffen Heegaard, Helle Broholm, Lasse S. Kristensen, Elisabeth Ralfkiaer, Kirsten Grønbæk

Corresponding author:

Kirsten Grønbæk
Professor, MD, DMSc.
Department of Hematology,
Rigshospitalet, Copenhagen University Hospital
Dept. 3733, Copenhagen Biocenter
Building 2, 3rd floor
Ole Maaløes Vej 5
2200 Copenhagen N
Denmark
Phone + 4535456086
Email: kirsten.groenbaek@regionh.dk

Additional Table 1a. Baseline characteristics of 107 PCNSL patients subjected to *TP53* mutational analysis, and *MIR34A* and *DAPK* promoter methylation studies

	All Patients	TP53 mutational analysis	MIR34a methylation analysis	DAPK methylation analysis
	N = 107 (100%)	N = 86 (80.4%)	N = 93 (86.9%)	N = 75 (70.1%)
**Sex**				
**Male, n (%)**	61 (57.0)	46 (53.4)	50 (53.8)	40 (53.3)
**Female, n (%**	46 (43.0)	40 (46.5)	43 (46.2)	35 (46.7)
**Age**				
**Mean (SE)**	65.3 (1.1)	65.2 (1.1)	64.8 (1.2)	64.2 (1.2)
**Median (min-max)**	66.0 (33-85)	66.0 (33-84)	66.0 (33-85)	65.0 (33-84)
**<65 years, n (%)**	51 (47.7)	40 (46.5)	45 (48.4)	37 (49.3)
**>65 years, n (%)**	56 (52.3)	46 (53.5)	48 (51.6)	38 (50.7)
**LDH[1]**				
**Mean (SE)**	268.4 (16.7)	266.2 (16.8)	262.1 (15.5)	267.8 (18.5)
**WHO performance status[2], n (%)**				
**Low (0-1)**	58 (59.8)	47	52	40
**High (2-4)**	39 (40.2)	30	34	28
**IPI-score[3]**				
**Low (0-1)**	34 (37.4)	28	30	27
**High (2-5)**	57 (62.6)	44	50	35
**Treatment**				
**CCT[4]**	70 (65.4)	57 (66,3)	62 (66.7)	50 (66.6)
**WBRT[5] monotherapy**	5 (3.7)	5 (5.8)	5 (5.4)	5 (6.7)
**HDMTX only[6]**	13 (12.1)	9 (10.5)	12 (12.9)	7 (9.3)
**No therapy**	19 (17.8)	15 (17.4)	14 (15.1)	13 (17.3)
**WBRT total[7]**				
**No**	70 (65.4)	57 (66.3)	62 (66.7)	48 (64.0)
**Yes**	37 (34.6)	29 (33.7)	31 (33.3)	27 (36.0)
**Total radiation dose, Gy**				
**Mean (SE)**	35.2 (1.3)	35.9 (1.2)	35.7 (1.2)	35.3 (1.3)
**Median (min-max)**	39.6 (6-40)	39.6 (20-40)	39.6 (20-40)	39.6 (20-40)
**Number of fractions**				
**Mean (SE)**	16.9 (1.1)	17.9 (1.1)	17.9 (1.1)	17.4 (1.2)
**Median (min-max)**	(2-22)	22 (5-22)	22 (4-22)	22 (4-22)
**Dose pr fraction**				
**Mean (SE)**	2.5 (0.2)	2.2 (0.13)	2.6 (0.26)	2.3 (0.18)
**Median (min-max)**	1.8 (1.8-8.0)	1.8 (1.8-4.4)	1.8 (1.8-8.0)	1.8 (1.8-5.5)

[1]LDH data available in 94 patients
[2]WHO-score data available in 97 patients
[3]IPI data available in 91
[4]Combination chemotherapy includes CNSBONN (patients<65 years: highdose-methotrexate (HDMTX), cyt-arabine, thiotepa, +/- rituximab, and ASCT (autologous stem cell transplantation), patients>65 years: methotrexate, vincristine, procarbacine +/- rituximab), carmustine+HDMTX, CNS IELSG (CHOP/CHOP-like regimens +/-HDMTX, cytarabine or alkylating agents+methotrexate), NORDIC CNS (CHOP-like regimen: rituximab, HDMTX, highdose-cytarabine, cyclophosphamide, iphosphamide, vincristine, vindesine, followed by temozolomide, and intraspinal depocyte), MVBPCNS (HDMTX, vincristine, carmustine, prednisolone), vincristine+HDMTX, all +/- rituximab. One HDMTX-treated patient was also treated with rituximab (survived 1251 days). Totally, 70+1 patients 21/71 (29.6%) had rituximab. Of the whole cohort, 21/108 (19.4%) were treated with rituximab.
[5]Whole brain radiation therapy only
[6] HDMTX only = High-dose methotrexate only
[7] WBRT total = includes all patients that received WBRT at any point, i.e. as principal monotherapy or later as consolidation therapy following combination chemotherapy

Additional Table 1b. Baseline characteristics of 86 PCNSL patients with or without *TP53* mutation *(TP53* mutation vs *TP53* wild-type)

	All patients<br><br>N = 86 (100%)	*TP53* mutation<br><br>N = 32 (37.2%)	*TP53* wild-type<br><br>N = 54 (62.8%)	P-values
**Sex**				
**Male, n (%)**	46 (53.4)	16	30	P = 0.66
**Female, n (%)**	40 (46.5)	16	24	
**Age**				
**Mean (SE)**	65.2 (1.1)	64.7 (1.8)	65.5 (1.5)	P = 0.73
**Median (min-max)**	66.0 (33-84)	65.0 (34-80)	67.0 (33-84)	
**<65 years, n (%)**	40 (46.5)	17	23	P = 0.38
**>65 years, n (%)**	46 (53.5)	15	31	
**LDH[1]**				
**Mean (SE)**	266.2 (16.8)	274.7 (23.2)	260.1 (23.3)	P = 0.69
**WHO performance status[2], n (%)**				
**Low (0-1)**	47 (54.7)	18	29	P =1.0
**High (2-4)**	30 (45.3)	11	19	
**IPI-score[3]**				
**Low (0-1)**	28	11	17	P = 1,0
**High (2-5)**	44	18	26	
**Treatment**				
**CCT[4]**	57 (66.3)	24	33	P = 1.0
**WBRT[5] monotherapy**	5 (5.8)	0	5	
**HDMTX only[6]**	9 (10.5)	4	5	
**No therapy**	15 (17.4)	4	11	
**WBRT total[7]**				
**No**	57 (66.3)	37	20	P = 0.64
**Yes**	29 (33.7)	17	12	
**Total radiation dose, Gy**				
**Mean (SE)**	35.9 (1.2)	37.2 (1.8)	34.9 (1.5)	P = 0.32
**Median (min-max)**	39.6 (20-40)	39.6 (20-40)	39.6 (22-40)	
**Number of fractions**				
**Mean (SE)**	17.9 (1.1)	19.3 (1.6)	17.0 (1.4)	P = 0.31
**Median (min-max)**	22 (5-22)	22 (5-22)	20 (5-22)	
**Dose pr fraction**				
**Mean (SE)**	2.2 (0.13)	2.1 (0.20)	2.3 (0.18)	P = 0.57
**Median (min-max)**	1.8 (1.8-4.4)	1.8 (1.8-4.0)	1.9 (1.8-4.4)	

[1]LDH data available in 74 patients
[2]WHO-score data available in 78 patients
[3]IPI data available in 73 patients
[4]Combination chemotherapy includes CNSBONN (patients<65 years: highdose-methotrexate (HDMTX), cyt-arabine, thiotepa, +/- rituximab, and ASCT (autologous stem cell transplantation), patients>65 years: methotrexate, vincristine, procarbacine +/- rituximab), carmustine+HDMTX, CNS IELSG (CHOP/CHOP-like regimens +/-HDMTX, cytarabine or alkylating agents+methotrexate), NORDIC CNS (CHOP-like regimen: rituximab, HDMTX, highdose-cytarabine, cyclophosphamide, iphosphamide, vincristine, vindesine, followed by temozolomide, and intraspinal depocyte), MVBPCNS (HDMTX, vincristine, carmustine, prednisolone), vincristine+HDMTX, all +/- rituximab.
[5] Whole brain radiation therapy only = primary treatment
[6] HDMTX only = High-dose methotrexate only
[7] WBRT total = includes all patients that received WBRT at any point, i.e. as principal monotherapy or later as consolidation therapy following combination chemotherapy

AdditionalTable 1c. Baseline characteristics of 93 PCNSL patients with or without *MIR34a* methylation

	All Patients<br>N = 93 (100%)	Methylated *MIR34a*<br>N = 53 (57.0%)	Un-methylated *MIR34a*<br>N = 40 (43.0%)	P-values
**Sex**				
**Male, n (%)**	50 (53.8)	31	19	P = 0.30
**Female, n (%)**	43 (46.2)	22	21	
**Age**				
**Mean (SE)**	64.8 (1.2)	65.1 (1.5)	64.4 (1.8)	P = 0.79
**Median (min-max)**	66.0 (33-85)	67.0 (34-84)	63.5 (33-85)	
**<65 years, n (%)**	45 (48.4)	23 (43.4)	22 (55.0)	P = 0.30
**>65 years, n (%)**	48 (51.6)	30 (56.6)	18 (45.0)	
**LDH[1]**				
**Mean (SE)**	262.1 (15.5)	270.5 (24.5)	251.6 (16.9)	P = 0.55
**WHO performance status[2], n (%)**				
**Low (0-1)**	52 (60.5)	35	17	P = 0.014
**High (2-4)**	34 (39.5)	13	21	
**IPI-score[3]**				
**Low (0-1)**	30 (37.5)	19	11	P = 0.36
**High (2-5)**	50 (62.5)	26	24	
**Treatment**				
**CCT[4]**	62 (66.7)	35	27	P = 0.91
**WBRT[5] monotherapy**	5 (5.4)	3	2	
**HD-MTX only[6]**	12 (12.9)	6	6	
**No therapy**	14 (15.1)	9	5	
**WBRT total[7]**				
**No**	62 (66.7)	37	25	P = 0.51
**Yes**	31 (33.3)	16	15	
**Total radiation dose, Gy**				
**Mean (SE)**	35.7 (1.2)	34.3 (1.9)	37.3 (1.4)	P = 0.21
**Median (min-max)**	39.6 (20-40)	39.6 (20-40)	39.6 (22-40)	
**Number of fractions**				
**Mean (SE)**	17.9 (1.1)	15.2 (1.8)	19.0 (1.4)	P = 0.11
**Median (min-max)**	22 (4-22)	20 (5-22)	22 (4-22)	
**Dose pr fraction**				
**Mean (SE)**	2.6 (0.26)	2.9 (0.40)	2.2 (0.25)	P = 0.17
**Median (min-max)**	1.8 (1.8-8.0)	1.8 (1.8-8.0)	1.8 (1.8-5.5)	

[1]Data available in 87 patients, of which 46 were methylated, and 32 were un-methylated

[2]Data available in 86 patients, of which 48 were methylated, and 38 were un-methylated

[3]Data available in 80 patients, of which 45 were methylated, and 35 were un-methylated

[4]Combination chemotherapy includes CNSBONN (patients<65 years: highdose-methotrexate (HDMTX), cyt-arabine, thiotepa, +/- rituximab, and ASCT (autologous stem cell transplantation), patients>65 years: methotrexate, vincristine, procarbacine +/- rituximab), carmustine+HDMTX, CNS IELSG (CHOP/CHOP-like regimens +/-HDMTX, cytarabine or alkylating agents+methotrexate), NORDIC CNS (CHOP-like regimen: rituximab, HDMTX, highdose-cytarabine, cyclophosphamide, iphosphamide, vincristine, vindesine, followed by temozolomide, and intraspinal depocyte), MVBPCNS (HDMTX, vincristine, carmustine, prednisolone), vincristine+HDMTX, all +/- rituximab.

[5] Whole brain radiation therapy only = primary treatment

[6]HDMTX only= High-dose methotrexate only

[7]WBRT total = includes all patients that received WBRT at any point, i.e as principal monotherapy or later as consolidation therapy, e.g. following combination chemotherapy

Additional Table 1d. Baseline characteristics of 75 PCNSL patients with or without *DAPK* methylation

	All Patients<br>N = 75 (100%)	Methylated *DAPK*<br>N = 70 (93.3%)	Un-methylated *DAPK*<br>N = 6 (6.7%)	P-values
**Sex**				
**Male, n (%)**	40 (53.3)	38	2	P = 0.66
**Female, n (%)**	35 (46.7)	32	3	
**Age**				
**Mean (SE)**	64.2 (1.2)	64.7 (1.2)	57.6 (7.7)	P = 0.15
**Median (min-max)**	65.0 (33-84)	65.5 (34-84)	58.0 (33-81)	
**<65 years, n (%)**	37 (49.3)	33	4	P = 0.20
**>65 years, n (%)**	38 (50.7)	37	1	
**LDH[1]**				
**Mean (SE)**	267.8 (18.5)	269.8 (19.9)	244.4 (39.7)	P = 0.72
**WHO performance status[2], n (%)**				
**Low (0-1)**	40 (58.8)	37	3	P = 1.0
**High (2-4)**	28 (41.2)	25	2	
**IPI-score[3]**				
**Low (0-1)**	27 (43.5)	25	2	P = 1.0
**High (2-5)**	35 (56.5)	32	3	
**Treatment**				
**CCT[4]**	50 (66.6)	46	4	P = 0.34
**WBRT[5] monotherapy**	5 (6.7)	4	1	
**HDMTX only[6]**	7 (9.3)	7	0	
**No therapy**	13 (17.3)	13	0	
**WBRT total[7]**				
**No**	48 (64.0)	46	2	P = 0.34
**Yes**	27 (36.0)	24	3	
**Total radiation dose, Gy**				
**Mean (SE)**	35.3 (1.3)	35.1 (1.5)	36.4 (3.2)	P = 0.77
**Median (min-max)**	39.6 (20-40)	39.6 (20-40)	36.9 (30-39.6)	
**Number of fractions**				
**Mean (SE)**	17.4 (1.2)	17.1 (1.4)	19.7 (2.3)	P = 0.52
**Median (min-max)**	22 (4-22)	22 (4-22)	22 (15-22)	
**Dose pr fraction**				
**Mean (SE)**	2.3 (0.18)	2.4 (0.20)	1.9 (0.07)	P = 0.38
**Median (min-max)**	1.8 (1.8-5.5)	1.8 (1.8-5.5)	1.9 (1.8-2.0)	

[1]Data available in 64 patients, of which 59 were methylated, and 5 were un-methylated
[2]Data available in 67 patients, of which 62 were methylated, and 5 were un-methylated
[3]Data available in 62 patients, of which 57 were methylated, and 5 were un-methylated
[4]Combination chemotherapy includes CNSBONN (patients<65 years: highdose-methotrexate (HDMTX), cyt-arabine, thiotepa, +/- rituximab, and ASCT (autologous stem cell transplantation), patients>65 years: methotrexate, vincristine, procarbacine +/- rituximab), carmustine+HDMTX, CNS IELSG (CHOP/CHOP-like regimens +/-HDMTX, cytarabine or alkylating agents+methotrexate), NORDIC CNS (CHOP-like regimen: rituximab, HDMTX, highdose-cytarabine, cyclophosphamide, iphosphamide, vincristine, vindesine, followed by temozolomide, and intraspinal depocyte), MVBPCNS (HDMTX, vincristine, carmustine, prednisolone), vincristine+HDMTX, all +/- rituximab.
[5]Whole brain radiation therapy only = primary treatment
[6]HDMTX only= High-dose methotrexate only
[7]WBRT total = includes all patients that received WBRT at any point, i.e as principal monotherapy or later as consolidation therapy, e.g. following combination chemotherapy

Additional Table 2a. Disease status of 107 PCNSL patients according to 1st line treatment.

Type of 1st line treatment/Status	Combination chemotherapy (n=70)	Whole brain radiation therapy (n=5)	Highdose-methotrexate only (n=13)	No therapy (n=19)
**Alive (n=23)**	**20**	**1**	**2**	**0**
**CR[1] after 1st line therapy**	12	-	2	-
**CRu[2] after 1st line therapy**	4	-	-	-
**Relapse after 1st line therapy**	(2)	1	-	-
**PR[3] after 1st line therapy**	4			
**Deceased (n=84)**	**50**	**4**	**11**	**19**
**Relapse after 1st line therapy (received 2nd treatment)**	17	0	3	0
**CR after 1st line therapy**	(3)	-	(1)	-
**CRu after 1st line therapy**	(9)		(1)	
**PR after 1st line therapy**	-	-	-	-
**PD[4]**	(4)	-	(1)	-
**SD[5]**	(1)	-	-	-
**PD (no 2nd treatment)**	32	4	7	19
**Therapy-related causes with PD**	(1)	-	(1)	-
**Unknown**	1	-	-	-

[1]CR: Complete remission
[2]CRu: Complete remission, unconfirmed
[3]PR: Partial remission
[4]PD: Progression of disease
[5]SD: Stable disease

Additional Table 2b. Table of PCNSL patients suffering a relapse, n=22/107 (20.6%), and their 2nd treatment. At follow-up 20 of these 22 patients had deceased and 2 were in complete remission.

2nd Treatment at Relapse	Number of patients n = 22
**Whole brain radiation therapy[§]**	7
**+ HDAraC[1] + rituximab**	1
**+ HDAraC, BCNU[2], rituximab**	1
**Combination chemotherapy[3¥]**	
**+ rituximab**	2
**- rituximab**	1
**Methotrexate[#]**	3
**Intrathecal therapy, unspecified[†]**	2
**No therapy[‡]**	4
**No data[₣]**	1

[1]Highdose cytarabine
[2]BCNU=carmustine
[3]CHOP, CNSBONN, MVBPCNS (for abbreviations, see footnotes tables 1a-d)
[§]Prior (1st) treatment before relapse; 4 patients were treated with combination chemotherapy (CCT, see footnotes tables 1a-d), 4 patients with CCT + rituximab, and 1 with HDMTX only
[¥, #, †]Prior (1st) treatment before relapse; totally, all 8 patients had CCT
[‡]Prior (1st) treatment before relapse; 3 patients had CCT and 1 patient had HDMTX
[₣]Prior (1st) treatment before relapse; 1 patient had HDMTX

Additional Table 3a. Immunohistochemical marker expression in 107 PCNSL patients

	Number of patients<br>N= 107<br>n (%)
**p53, n = 106**	
+	92 (86.8)
–	14 (13.2)
**MIB proliferation index, n = 89**	
**Mean (SE)**	78.9 (1.8)
**Median (min-max)**	80 (20-100)
**BCL2, n = 79**	
+	64 (62.3)
–	15 (37.7)
**BCL6, n = 67**	
+	50 (74.6)
–	17 (25.4)
**CD10, n = 104**	
+	19 (18.3)
–	85 (81.7)
**MUM1, n = 61**	
+	59 (96.7)
–	2 (3.3)
**HANS Classification, n = 70**	
**GCB**	20 (28.6)
**Non-GCB**	50 (71.4)

Additional Table 3b. Immunohistochemical marker expression in 86 patients with PCNSL screened for *TP53* mutation. Presence of expression is designated as a "+" and absence as a "–".

	Number of patients<br><br>N = 86	*TP53* mutation<br><br>n = 32 (32.7%)	*TP53* wild-type<br><br>n = 54 (67.3%)	P-values
**p53, n = 86**				
+	76 (88.4)	26	50	P = 0.73
–	10 (11.6)	4	6	
**MIB proliferation index, n = 75**				
**Mean (SE)**	80.2 (1.8)	73.9 (3.8)	83.8 (1.7)	P = 0.008
**Median (min-max)**	80 (20-100)	75 (20-95)	85 (40-100)	
**BCL2, n = 63**				
+	51 (81.0)	19	32	P = 0.52
–	12 (19.0)	3	9	
**BCL6, n = 55**				
+	40 (72.7)	14	26	P = 0.54
–	15 (27.3)	7	8	
**CD10, n = 84**				
+	12 (14.5)	3	9	P = 0.52
–	71 (85.5)	27	44	
**MUM1, n = 50**				
+	49 (98.0)	17	32	P = 1.0
–	1 (2.0)	0	1	
**HANS Classification, n = 56**				
**GCB**	13 (23.2)	3	10	P = 0.33
**Non-GCB**	43 (76.8)	18	25	

Additional Table 3c. Immunohistochemical marker expression in 78 of 93 with PCNSL screened for *MIR34A* promoter methylation. Presence of expression is designated as a ”+”, and absence as a ”–”.

	**Number of patients**	***MIR34A* methylated**	***MIR34A* un-methylated**	**P-values**
	**N = 78**	**n = 46 (59.0%)**	**n = 32 (41.0%)**	
**MIB Proliferation Index, n = 78**				
**Mean (SE)**	80.3 (1.8)	81.1 (2.0)	77.9 (3.5)	P = 0.30
**Median (min-max)**	82.5 (20-100)	85 (20-100)	80 (30-95)	
**BCL2, n = 69**				
+	57 (82.6)	36	21	P = 0.52
–	12 (17.4)	6	6	
**BCL6, n = 61**				
+	45 (73.8)	31	14	P = 0.23
–	16 (26.2)	8	8	
**CD10, n = 91**				
+	17 (18.7)	11	6	P = 0.59
–	74 (81.3)	40	34	
**MUM1, n = 56**				
+	54 (96.4)	36	18	P = 1.0
–	2 (3.6)	1	1	
**HANS classification, n = 64**				
**GCB**	18 (28.1)	12	6	P = 0.78
**Non-GCB**	46 (71.9)	28	18	

Additional Table 3d. Immunohistochemical marker expression in 76 patients with PCNSL screened for *DAPK* promoter methylation. Presence of expression is designated as a "+", and absence as a "–".

	Number of patients<br>N=75	*DAPK* methylated<br>N= 70 (93.3%)	*DAPK* Un-methylated<br>N= 5 (6.7%)	P-values
**MIB Proliferation Index, n = 78**				
**Mean (SE)**	81.1 (1.9)	80.8 (2.0)	85.0 (5.8)	P = 0.30
**Median (min-max)**	80.0 (20-100)	80.0 (20-100)	85.0 (75-95)	
**BCL2, n = 56**				
+	45 (80.4)	45	0	P = 0.036
–	11 (19.6)	9	2	
**BCL6, n = 49**				
+	35 (71.4)	33	2	P = 1.0
–	14 (28.6)	13	1	
**CD10, n = 74**				
+	12 (16.2)	10	2	P = 0.25
–	62 (83.8)	58	4	
**MUM1, n = 43**				
+	42 (97.7)	41	1	P = 1.0
–	1 (2.3)	1	0	
**HANS classification, n = 50**				
**GCB**	13 (26.0)	11	2	P = 0.16
**Non-GCB**	37 (74.0)	36	1	
