# Supplementary material for: TP53 hotspot mutations are predictive of survival in primary central nervous system lymphoma patients treated with combination chemotherapy
Source: Acta Neuropathol Commun. 2016 Apr 22;4:40. doi: 10.1186/s40478-016-0307-6 (PMC4840983; doi:10.1186/s40478-016-0307-6)
Supplement: Additional file 3: Figure S1a-c. — Supplemental figures 1a-c. Figure S2a-b. Supplemental figures. (PDF 474 kb) [file 40478_2016_307_MOESM3_ESM.pdf]

Title:
***TP53* hotspot mutations are predictive of survival in primary central nervous system lymphoma patients treated with combination chemotherapy**

Journal Name: Acta Neuropathologica Communications

Authors:
Helga D. Munch-Petersen, Fazila Asmar, Konstantinos Dimopoulos, Aušrinė Areškevičiūtė, Peter de Nully Brown, Mia Seremet Girkov, Anja Pedersen, Lene D. Sjö, Steffen Heegaard, Helle Broholm, Lasse S. Kristensen, Elisabeth Ralfkiaer, Kirsten Grønbæk

Corresponding author:
Kirsten Grønbæk
Professor, MD, DMSc.
Department of Hematology,
Rigshospitalet, Copenhagen University Hospital
Dept. 3733, Copenhagen Biocenter
Building 2, 3rd floor
Ole Maaløes Vej 5
2200 Copenhagen N
Denmark
Phone + 4535456086
Email: kirsten.groenbaek@regionh.dk

Additional figure 1a-b

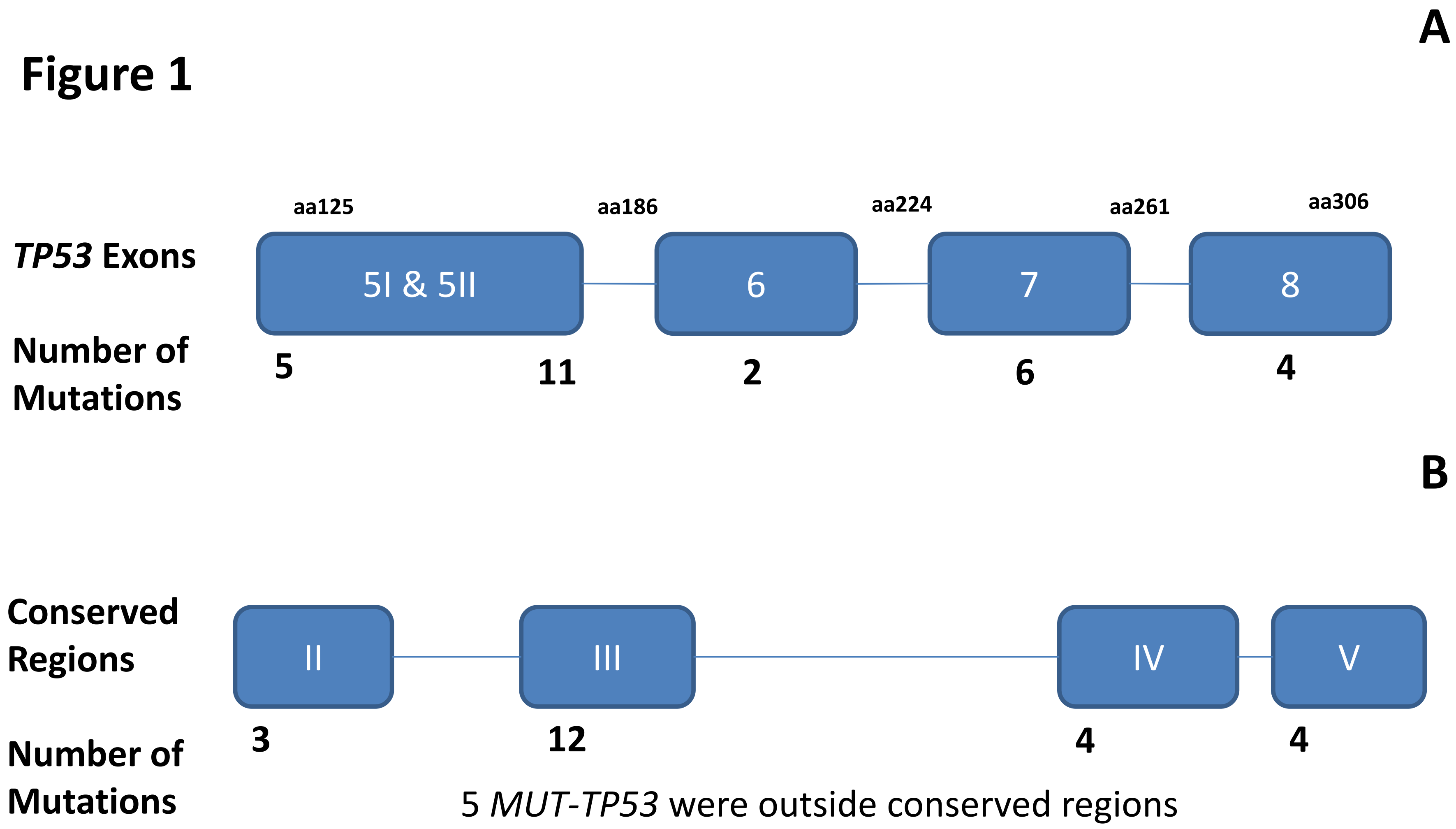

Additional figure 1C

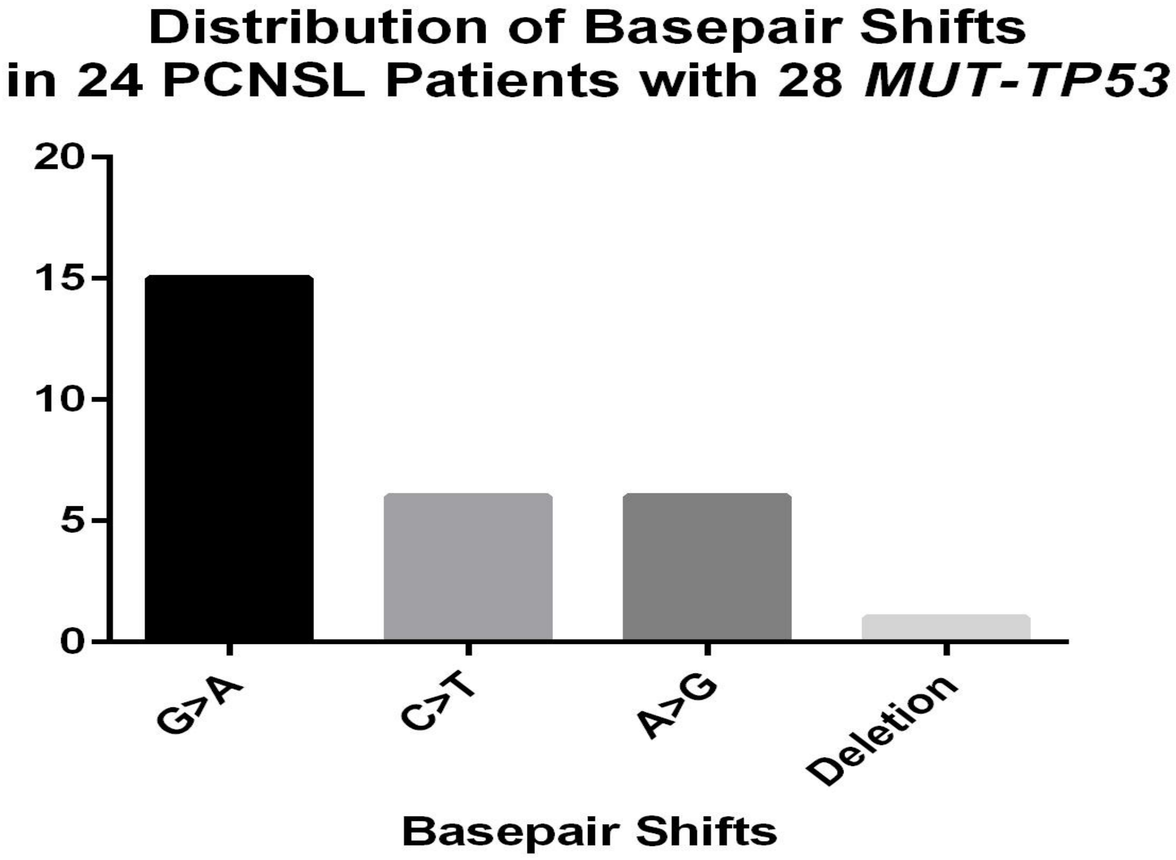

Additional figure 2a-b

A

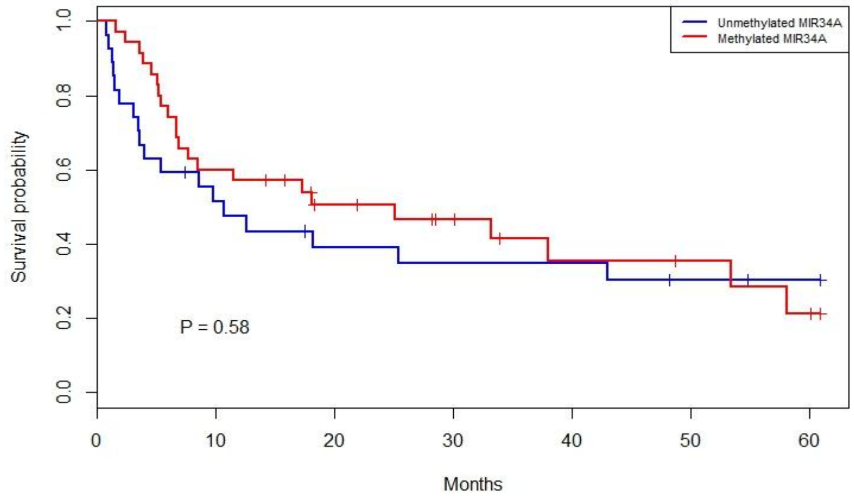

B

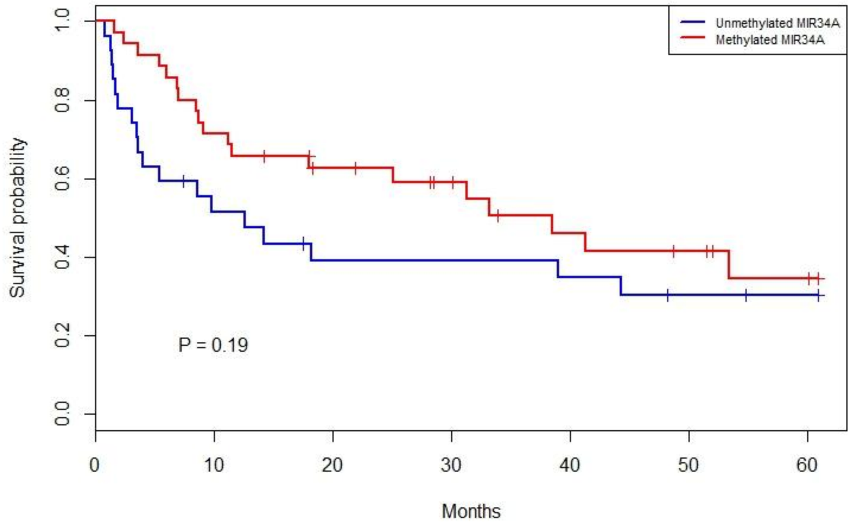

Additional figure legends

Additional Figure 1

Structural characteristics of 28 *MUT-TP53* (n=24) in CCT-treated PCNSL patients

According to **A**: distribution in exons 5-8, **B**: conserved regions 2-5, and **C**: proportion of basepair changes of *MUT-TP53*. G>A: 15 (53.6 %), C>T: 6 (21.4%), A>G: 6 (21.4%), del: 1 (3.6%).

Additional Figure 2

***MIR34A* methylation status and survival in CCT-treated PCNSL patients (n=57)**

Kaplan-Meier plot of **A**: PFS, and **B**: OS. There was no difference in survival among patients with or without *MIR34A* methylation, P = 0.58 and P = 0.19, respectively.
