# Supplementary material for: TP53 hotspot mutations are predictive of survival in primary central nervous system lymphoma patients treated with combination chemotherapy
Source: Acta Neuropathol Commun. 2016 Apr 22;4:40. doi: 10.1186/s40478-016-0307-6 (PMC4840983; doi:10.1186/s40478-016-0307-6)
Supplement: Additional file 4: — Data methylation status and survival. (PDF 262 kb) [file 40478_2016_307_MOESM4_ESM.pdf]

Title:

TP53 hotspot mutations are predictive of survival in primary central nervous system lymphoma patients treated with combination chemotherapy

Journal Name: Acta Neuropathologica Communications

Authors:

Helga D. Munch-Petersen, Fazila Asmar, Konstantinos Dimopoulos, Aušrinė Areškevičiūtė, Peter de Nully Brown, Mia Seremet Girkov, Anja Pedersen, Lene D. Sjö, Steffen Heegaard, Helle Broholm, Lasse S. Kristensen, Elisabeth Ralfkiaer, Kirsten Grønbæk

Corresponding author:

Kirsten Grønbæk

Professor, MD, DMSc.
Department of Hematology,

Rigshospitalet, Copenhagen University Hospital

Dept. 3733, Copenhagen Biocenter
Building 2, 3rd floor
Ole Maaløes Vej 5
2200 Copenhagen N
Denmark

Phone + 4535456086

Email: kirsten.groenbaek@regionh.dk

Additional Data 1

Survival According to rs13300553 SNP Genotypes & Allellic *DAPK* Methylation Patterns

Genotyping by Sanger sequencing of the rs13300553 SNP was successful in 78/107 of the samples. Thirty-seven samples were genotyped as AG, 25 as AA and 16 as GG. No relationship between individual genotypes (AG, AA, GG) and OS was observed, $P = 0.810$.

Pyrosequencing was performed to assess the allelic methylation patterns in the 37/78 (%) patients that were heterozygous. This approach was successful in 34/37 (%) of the samples. Twenty-six had a biallellic methylation pattern, and 2 had a monoallellic methylation pattern on the G-allele (defined as C-allele below 20%), and 3 on the A-allele (defined as T-allele below 20%), respectively, while 3 were unmethylated. There was no relationship between OS and individuals with bi- versus monoallellic methylation patterns, $P = 1.00$.
